# Optimization of the Extraction of Bioactive Compounds and Metabolomic Profile of *Licaria armeniaca*

**DOI:** 10.3390/plants14203158

**Published:** 2025-10-14

**Authors:** Lanalice R. Ferreira, Bianca R. Abelém, José Diogo E. Reis, Christelle Anne N. P. Herman, Pablo Luis B. Figueiredo, Laine Celestino Pinto, Luiza Helena Martins, Milton Nascimento da Silva, Paulo Wender P. Gomes, Joyce Kelly R. da Silva

**Affiliations:** 1 Programa de Pós-Graduação em Química, Universidade Federal do Pará, Belém 66075-110, PA, Brazil; lanalice.ferreira@icen.ufpa.br (L.R.F.); reis.diogo190@gmail.com (J.D.E.R.); yumilton@yahoo.com.br (M.N.d.S.); wendergomes@ufpa.br (P.W.P.G.); 2 Faculdade de Biotecnologia, Universidade Federal do Pará, Belém 66075-110, PA, Brazil; biancaablem@gmail.com (B.R.A.); christelle.herman.pompeu@gmail.com (C.A.N.P.H.); 3 Central Integrada em Metabolômica da Amazônia, Instituto de Ciências Exatas e Naturais, Universidade Federal do Pará, Belém 66075-110, PA, Brazil; 4 Laboratório de Química dos Produtos Naturais, Universidade do Estado do Pará, Belém 66095-015, PA, Brazil; pablo.figueiredo@uepa.br; 5 Laboratório de Neuropatologia Experimental, Hospital Universitário João de Barros Barreto, Universidade Federal do Pará, Belém 66073-000, PA, Brazil; lainecelestino@hotmail.com; 6 Programa de Pós-Graduação em Biotecnologia Aplicada à Agricultura, Universidade Federal Rural da Amazônia, Belém 66077-830, PA, Brazil; luiza.martins@ufra.edu.br; 7 Programa de Pós-Graduação em Biodiversidade e Biotecnologia (BIONORTE-Pará), Universidade Federal do Pará, Belém 66075-110, PA, Brazil

**Keywords:** response surface methodology, green extraction, total phenolic content, antioxidant activity, LC–MS analysis, molecular networking

## Abstract

The ultrasound-assisted extraction (UAE) method was optimized to extract bioactive compounds from *Licaria armeniaca* tissues. Extraction time, solid–liquid ratio (*m*/*v*), and ethanol percentage were investigated using a central composite rotational design and response surface methodology (RSM). Antioxidant activity (DPPH) and total phenolic content (TPC) served as the response variables. Most efficient extraction conditions were obtained for leaves (64.88% ethanol, 26.07 min, 6.23% *m*/*v*; R^2^ = 0.93) and thin branches (73.81% ethanol, 31.34 min, 11% *m*/*v*; R^2^ = 0.74). For thick branches, no significant predictive model was obtained, and optimal points were defined based on the best observed TPC and DPPH results (50% ethanol, 35 min, 11% *m*/*v*). The optimized extracts were analyzed by liquid chromatography–tandem mass spectrometry associated with molecular networking, GNPS (Global Natural Products Social Molecular Network) library searching, and machine learning tools. Metabolomic profiling indicated that leaves contained mainly alkaloids (46.34%), amino acids and peptides (19.51%), and shikimate derivatives and phenylpropanoids (12.20%). Thin branches showed predominance of alkaloids (35.97%), amino acids and peptides (20.86%), and carbohydrates (12.23%), while thick branches contained alkaloids (46.34%), amino acids and peptides (25.00%), and fatty acids (14.26%). Additionally, the extracts displayed significant cytotoxic activity against cancer cell lines of AGP-01 (malignant gastric ascites), AHOL (Human glioblastoma) and A549 (lung cancer) with IC50 values less than 50 μg/mL.

## 1. Introduction

*Licaria* is a Neotropical genus that occurs in the Cerrado (1 spp.), Atlantic Forest (6 spp.), and Amazon (23 spp.) biomes of Brazil [1]. *Licaria armeniaca* is a tree distributed in the Amazon Rainforest, popularly known as laurel, yellow-flowered laurel, or purple laurel [2,3]. Chemical studies on its composition were published in the 1970s and 1980s on extracts obtained by percolation with the solvents as hexane, benzene, and ethanol from wood and fruits, reporting the presence of alkaloids, coumarins, steroids, lignans, and neolignans [4,5,6,7]. These compounds have potential technological uses associated with their biological activities, such as antioxidants, anti-inflammatory, and anticancer effects [8,9,10,11].

The extraction process is considered the first step in the isolation and identification of bioactive compounds. Therefore, we highlight ultrasound-assisted extraction (UAE), explored as a green, innovative, economical, and sustainable technology [12,13]. The technique provides higher yields in obtaining bioactive compounds from plant sources, in addition to reducing extraction time, solvent use, and energy consumption [14,15]. The recovery of phenolic compounds extracted from *Nectandra grandiflora* (Lauraceae) obtained the highest phytochemical contents and antioxidant activity achieved by conventional extraction; however, they did not present major differences in alternative ecological techniques, such as UAE [16].

The efficiency of UAE is influenced by several factors in the extraction process, including the type of solvent used, extraction time, solid–liquid ratio, pH level, and temperature [17]. Therefore, it is important to establish optimal experimental conditions for each plant sample to maximize the yield of bioactive compounds [18]. Under these conditions, the proper choice of a solvent is essential to the process. Thus, ethanol stands out as an efficient solvent, being characterized as a green solvent, as it is produced from sustainable sources, is biodegradable, safe, easily recoverable, making it a viable alternative to traditional organic solvents in the extraction of bioactive compounds [19,20]. To account for these variables, the Central Composite Rotational Design (CCRD) experimental framework is utilized. This approach aims to optimize the results of experiments while minimizing the number of trials required [21,22].

CCRD experimental design is often combined with the response surface methodology (RSM), which uses statistical methods applied in research and aims to establish ideal conditions and broaden the understanding of the nature of certain phenomena [23]. However, one of the problems observed in the use of experimental design is dealing with multiple responses in a single analysis. Thus, the Global Desirability Function (d) can be applied to simultaneously determine the optimum point of a set of variables that can determine the best performance of one or more responses [24,25,26].

Complementing the UAE technique with experimental design has been used to optimize the production of phenolic compounds. In *Laurus nobilis* (Lauraceae), the use of these methodologies with different experimental conditions indicated that extracts with high amounts of phenolic compounds were extracted by UAE [27].

To investigate plant metabolites, liquid chromatography coupled with tandem mass spectrometry (LC-MS/MS) combined with machine learning tools, such as the Molecular Network available at Global Natural Products Social Molecular Networking (GNPS), has been widely used to identify the chemical diversity of plants [28], providing useful information for structural characterization [29,30]. Thus, the objective of this work was to evaluate the ultrasound-assisted extraction of bioactive compounds from *L. armeniaca*, systematically optimized based on the CCRD-type experimental design, use of RSM and Harrington’s global desirability function (d).

## 2. Results and Discussion

### 2.1. Optimization of the Extraction of Bioactive Compounds

**Figure 1:**
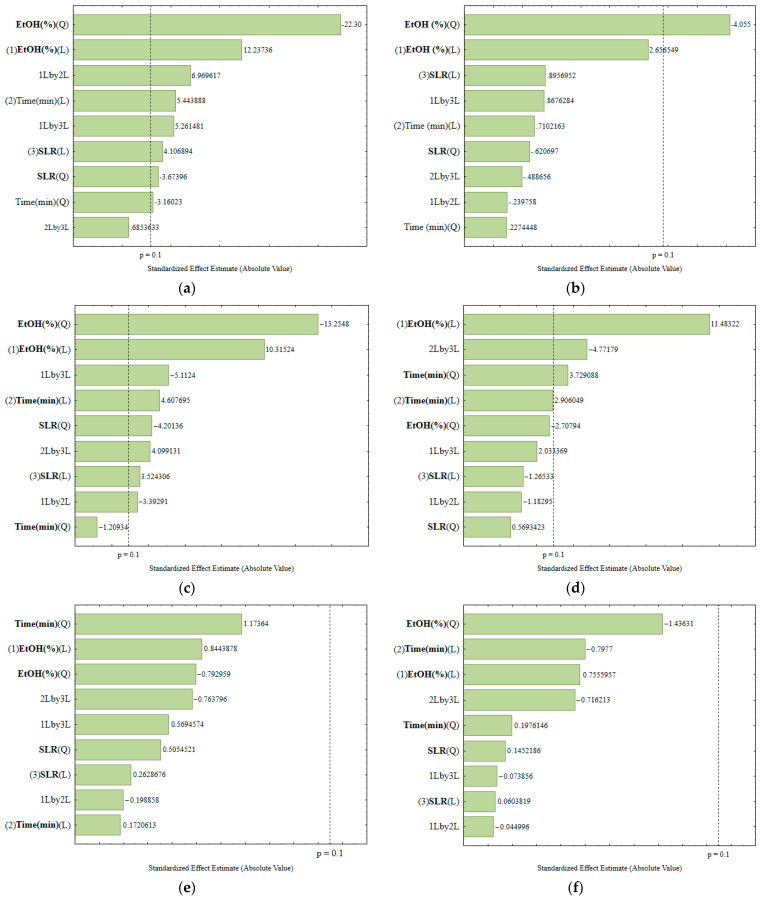
Pareto chart of effects estimates for DPPH (%) (**a**) leaves, (**c**) thin branches, (**e**) thick branches; TPC (mg GAE/g); (**b**) leaves, (**d**) thin branches and (**f**) thick branches. Source: Obtained with Software Statistics 7.0.

The results of the statistical analysis of the process variables for the extraction of phenolic compounds from *L. armeniaca* extracts are presented in Pareto charts (Figure 1a–f), obtained with 90% confidence. Statistically significant effects are located to the right of the significance limit for *p* < 0.1. With the analysis of the graphs, it was possible to visually understand the effects of the independent variables and their interpretations.

In Figure 1a, referring to the DPPH (%) obtained from the leaves, we observe significant effects of linear and quadratic variables, and possibly some interactions. This result underscores the intricate nature of the extraction process, where linear variables explain the direct increase in response as, for example, tempo or ethanol percentage in-creases. Quadratic effects, on the other hand, reflect the existence of an optimum point: at very low or very high concentrations, the yield of antioxidant activity decreases, which is typical for unstable phenolic compounds. Significant interactions, such as time versus ethanol percentage, reveal that the factors do not act in isolation and that the impact of one depends on the level of the other, characterizing the complexity of the extraction process for the leaves.

In Figure 1b, which shows the TPC (mg GAE/g) for the leaves, the configuration of significant effects differs from that observed for DPPH. Here, only the variable ethanol percentage, the quadratic effect, cross the line of significance. This suggests that the extraction of total phenolic compounds does not depend on the same set of variables that determine antioxidant activity. The unique behavior of the two responses in the same type of plant matrix highlights the diversity of our research. In many cases, phenolic solubility is more influenced by solvent than by time, which explains the difference in behavior between the two responses.

Figure 1c, referring to DPPH (%) in thin twigs, a change in the pattern of significance is observed. Unlike leaves, this matrix contains compounds in smaller quantities and is more strongly associated with the cell wall structure. Thus, quadratic factors such as temperature may appear more important, indicating a narrow stability limit for the anti-oxidants present in thin twigs. The fact that some variables lose significance in this matrix reflects the distinct chemical composition, where the availability of antioxidant compounds is lower and more difficult to release.

Figure 1d, for TPC in thin twigs, it can be seen that the response presents fewer significant variables, with only one or two, or even none, emerging. This result suggests that the amount of phenolics extracted from this matrix is relatively low and does not respond strongly to the experimental conditions tested. In other words, even if the ethanol per-centage, time, or solid-to-liquid ratio is changed, the extraction of phenolics from thin branches is limited by the sample composition itself, which explains the reduced pattern of significance.

In Figure 1e, which corresponds to the DPPH (%) in thick branches, the trend is for even fewer significant effects to appear, since this matrix has a lower concentration of secondary metabolites available for extraction. In this case, when a quadratic effect is observed, it generally reflects the difficulty in optimizing the release of antioxidant compounds, indicating that extreme increases in the percentage of ethanol or in time do not favor the process. The lack of significance in some variables shows that, for thick branches, the antioxidant potential is less dependent on operating conditions and more conditioned by the limited content of active compounds.

Finally, in Figure 1f, corresponding to the TPC in thick branches, the scenario is similar to that in the previous figure. The number of significant variables tends to be small, and often only the solvent or an isolated interaction stands out. This can occur because the phenolic compounds in this matrix are more closely bound to the lignocellulosic structure, hindering efficient extraction. Thus, even variables that proved relevant in leaves and thin twigs fail to exert a significant impact here, revealing that the chemical composition of the matrix and the low availability of phenolic compounds are the main limiting factors in the process.

The significance of variables such as ethanol percentage, solid-to-liquid ratio, and ex-traction time, as well as their interactions, differs among leaves, fine branches, and coarse branches due to structural and compositional differences. Leaves, with higher surface area and less lignified tissue, respond more to changes in extraction conditions, while denser branch tissues limit solvent penetration, making some effects non-significant [31]. In the optimization of the extraction of phenolic compounds from *Laurus nobilis* (Laureceae) by ultrasound-assisted extraction, the Pareto analysis evidenced the solid/liquid ratio and ethanol concentration as the most significant variables (*p* < 0.05) in the recovery of phenolic compounds [27].

ANOVA analysis and Test of Fisher were performed only on Leaves (DPPH—Y_1_^F^) and Thin Branches (DPPH and TPC–Y_2_^GF^), as they present significant effects, as shown in Table 1.

From the statistical analysis, two mathematical models considered significant and predictive were obtained, which were adopted for subsequent studies: the antioxidant activity for leaf extracts (DPPH) (Y_1_^F^) and the total phenolic compound content for fine twig extracts (Y_2_^GF^). The antioxidant activity for fine twigs was not considered significant and predictive.

In the leaf extraction, three linear effects, three quadratic effects and two interaction effects were significant in determining the DPPH, generating Equation (1). The coefficient of determination, close to 1 R^2^ = 0.93, reveals the ability of the equation to describe the real behavior. With F_calc_ > F_tab_ and a non-significant Lack of fit (*p*-value > 0.1), the adequacy of the model was ensured:

Y_1_^F^ = 65.26 + 5.92(X_1_) + 2.63(X_2_) + 1.99 (X_3_) − 11.89(X_1_^2^) − 1.68(X_2_^2^) − 1.96(X_3_^2^) + 4.4 (X_1_X_2_) + 3.32(X_1_X_3_) (1)

Regarding the extraction of thin branches, two linear effects and one interaction effect influenced TPC, adjusted in Equation (2). This model presented R_2_ = 0.74, indicating 74% of the variation in the observed data. The value of F_calc_ > F_tab_ and the non-significance of the lack of adjustment (*p*-value > 0.1) indicate that the model is predictive for the description of the real response.

Y_2_^GF^ = 328.61 + 38.13(X_1_) + 13.64(X_2_) − 20.69(X_2_X_3_) (2)

TPC model for thin branches could explain only 74% in the explanatory power, and the same behavior was observed also for the thin branches in DPPH (59%), these values represent a moderate level of reliability, and this limitation can be justified because of the inherent variability of the biological material and the complexity of phenolic compound biosynthesis, which is influenced by multiple environmental and physiological factors not fully captured in the current model.

Regarding the DPPH value, this variation can be attributed to the diversity and concentration of phenolic compounds present, the interference of other constituents of the matrix and the sensitivity of the assay to experimental conditions, reflecting the chemical complexity of the samples analyzed.

Despite both these models being considered moderated, they still provide meaningful information into the relationship between TPC, DPPH and the parameters studied for the thin branches. To overcome this, future studies may improve model performance by including additional variables or alternative modeling approaches in this raw material exploration.

**Figure 2:**
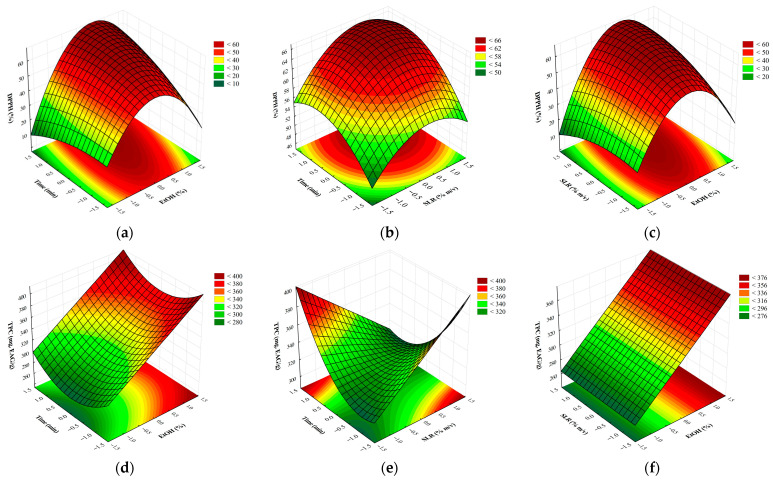
Response surface of *L. armeniaca* extracts. % DPPH inhibition leaves: (**a**) time versus %EtOH; (**b**) time versus SLR; (**c**) SLR versus %EtOH; and TPC—thin Branches: (**d**) time versus %EtOH; (**e**) time versus SLR; (**f**) SLR versus %EtOH. Source: Obtained with Statistica 7.0 Software.

The Response Surface Methodology (RSM) was applied to generate the graphs in Figure 2a–f, which will be discussed below:

Figure 2a–e show that the response surface graphs obtained presented a non-linear behavior of the studied variables, indicating that the relationship between the experimental factors and the responses does not follow a strictly proportional pattern. This non-linear behavior is common in bioactive compound extraction processes using UEA, since several interactions can occur between the variables, influencing the extraction efficiency in a complex way [32].

Figure 2a–c shows the tendency to reach a maximum region of extraction of the bioactive compounds analyzed. This behavior may suggest that the experimental variables may interact in a complex way, so that the continuous increase in a factor does not necessarily result in a continuous improvement of the response. To explain this phenomenon, we must consider the different physicochemical mechanisms involved in the extraction process.

Studies reinforce that, in processes of extraction of bioactive compounds from plants, interactions between variables such as type and concentration of solvent, temperature and extraction time are often non-linear on the responses, indicating complex interactions that influence extraction efficiency [33]. In view of this, the need for techniques such as experimental design and mathematical modeling to optimize extraction processes is reinforced [34,35]. Figure 2d–f indicate that high combinations between time and SLR disfavor the extraction of phenolic compounds in thin branches. The response surface reveals that the highest TPC values occur by maximizing one of the parameters and minimizing the other.

In short, the presence of maxima in the graphs from the experiments highlights the importance of optimizing the extraction conditions, since excess time, solvent or ethanol can compromise the efficiency of the process. This non-linear behavior attests to the importance of the response surface methodology to determine the optimal extraction conditions, aiming at the balance between yield and stability of the bioactive compounds.

### 2.2. Optimization by Desirability Tool

**Figure 3:**
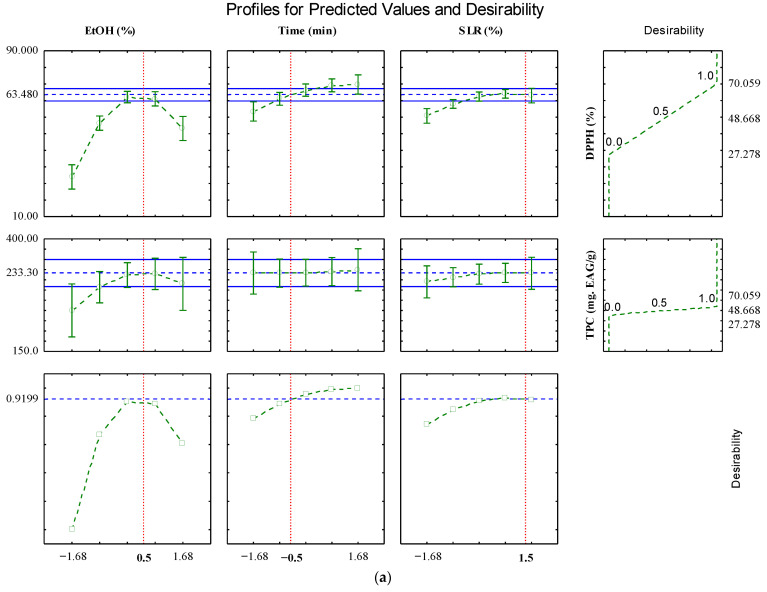

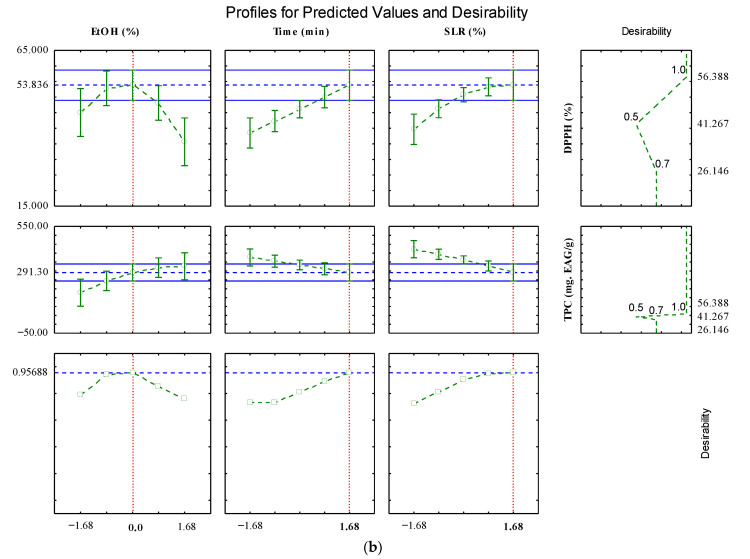
Profiles for predicted values and desirability for the optimization of ethanol percentage (%), extraction time (min) and solid–liquid ratio (% *w*/*v*) for bioactive extraction of *L. armeniaca* leaves using ultrasound-assisted extraction. (**a**) leaves; (**b**) thin branches.

After analyzing ANOVA and RSM and obtaining the mathematical models, we conducted an optimization analysis using the desirability tool. The resulting graphs, illustrated in Figure 3, aim to identify the optimal values for each variable, thereby enhancing the process optimization. The graphs depict the predicted behavior of the model, with the bottom lines indicating the best conditions based on desirability. The red lines highlight the optimal conditions for each variable individually.

The overall desirability value for the leaves test was 0.9199, indicating that our experimental data fit into a quite acceptable desirability range between 0.8 and 1.00 [24]. The values predicted by the model, as observed in Figure 3a were: X_1_ = 0.5 (65.4% EtOH), X_2_ = −0.5 (26.1 min) and X_3_ = 1.5 (7.6% *m*/*v*), which would result in Y_1_^F^ = 63.48% and Y_2_^F^ = 233.39. However, the model validation was carried out considering only the laboratory replicates of DPPH in the leaf samples. Once TPC did not present a significant effect in the ANOVA (Table 1) it was not considered in the experimental validation.

Regarding the thin branches in desirability, the overall desirability value was 0.957, indicating that our experimental data fit into a quite acceptable desirability range between 0.8 and 1.00 [24]. The values predicted by the model, as observed in Figure 3b: X_1_ = 0 (50.8% EtOH), X_2_ = 1.68 (65 min) and X_3_ = 1.68 (20% *m*/*v*) which would result in Y_2_^GF^ = 291.30 mgEAG/g, since DPPH did not present a significant effect in the ANOVA (Table 1) it was not used in the experimental validation.

These considered conditions found were subjected to the extraction process and the experimental values obtained were compared with the predicted ones, in order to confirm the reliability and predictive capacity of the models in this study.

The relative error between the experimental values and those predicted by the model was 1.78% for DPPH of leaves and 3.33% for TPC of thin branches (Table 1). Thus, mathematical model can be considered good for predicting the extraction of phenolic compounds and antioxidant compounds extracted from *L. armeniaca* using EtOH: H_2_O/UAE.

In this study was adopted a confidence level of 90% (*p* < 0.1) for the comparison between experimental and predicted values, once spectrophotometric analyses are inherently variable (Table 2). This relatively lower confidence level was chosen considering the natural fluctuations in the measurements, allowing for a realistic assessment of the agreement between predicted and experimental data. When observing the experimental values obtained, they were all within this adopted confidence limit.

### 2.3. Metabolomic Profile of L. armeniaca Extracts

**Figure 4:**
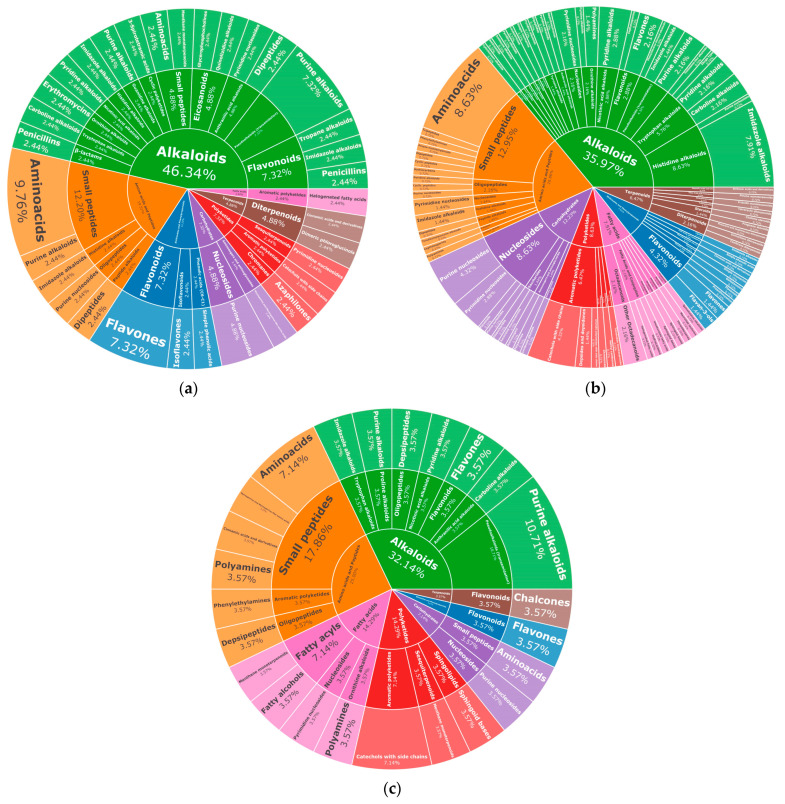
Metabolomic profile of optimized extracts of *L. armeniaca* tissues obtained by UEA. (**a**) leaves; (**b**) thin branches; (**c**) thick branches. The percentages indicate the relative proportion of each class calculated from the total ion signal intensity (TIC) of all detected features.

The compounds present in the optimized extracts of leaves, thin twigs, and thick twigs of *L. armeniaca* were analyzed by LC-MS/MS. A total of 100% of classes of metabolites was identified in the leaf extracts with predominance of alkaloids (46.34%), amino acids and peptides (19.51%), and shikimate and phenylpropanoid derivatives (12.20%). From a total of 98.98% of metabolites identified in the thin branches and of 100% identified in the thick branches, the major classes were alkaloids (35.97%), amino acids and peptides (20.86%), and carbohydrates (12.23%), and alkaloids (32.14%), amino acids and peptides (25.00%), and fatty acids (14.26%), respectively (Figure 4).

Alkaloids can be considered important biomarkers for the evolution of Lauraceae species [36]. They are widely reported in *Ocotea*, *Litsea*, *Cryptocarya*, and *Neolitsea*, predominantly as aporphine, benzylisoquinoline, and bisbenzylisoquinoline alkaloids [37,38]. Among the alkaloid types, aporphine types were reported in 22 of the 23 genera that represent the predominant group in Lauraceae [39]. In this study, the imidazole, pyridine, and purine subclasses were identified with higher occurrences, differing from previous studies of *Licaria*, which were predominantly benzylisoquinolines, proaporphines, and aporphines. Bacteolin, *O*-methylbracteolin (aporphine alkaloids) and α-dehydroreticuline (benzylisoquinoline alkaloid) were identified from extracts of *L. armeniaca* stem bark [6]. The occurrence of alkaloids was also reported for extracts of *L. puchury-major*, with an emphasis on benzylisoquinolines, such as reticuline, coclaurine, isoboldine, and norisoboldine, obtained from the seeds [40].

There are two classical strategies for extracting alkaloids: one involves using acidified water to extract them in the form of salts. In contrast, the other method consists of releasing them with a base and then extracting them with a low-polarity solvent [41]. However, modern methods, particularly ultrasound-assisted extraction (UAE), are effective for extracting alkaloids, largely because they do so in reduced time and solvent consumption [42,43]. Despite advancements in extraction techniques, there is still a lack of studies linking the effectiveness of unconventional extraction methods to the bioactivities of Lauraceae extracts.

The investigation of the extraction efficiency of alkaloids from *Coptidis chinensis* by UAE and heating reflux extraction highlighted that RSM was successfully implemented for optimization (optimal points for both techniques: 59% ethanol, 47 min extraction time at 66.22 °C). UAE increased both the total alkaloid content and the levels of berberine and palmatine. Furthermore, there was an increase in the antioxidant capacity due to the increased movement of solvent molecules caused by the sonication effect. Additionally, the UAE technique showed a higher yield (64.3%) compared to heating reflux (42.5%), which underestimated optimal UAE conditions [43].

A comparative study of alkaloid extraction techniques used ethanol: water (20:80, 40:60, 50:50, 60:40, 80:20, and 100:0, *v*/*v*), short extraction times (5, 10, and 15 min), and small sample amounts (10:1, 20:1, and 30:1 in 10 mL). The results demonstrated that ultrasound-assisted extraction (UAE) and microwave-assisted extraction (MAE) were as efficient as percolation (10 mg extracted with 100 mL dichloromethane). Furthermore, MAE was as efficient as UAE for the extraction of tetrahydropalmatine, and UAE significantly increased the extraction yields of palmatine and roemerine, alkaloids present in *Stephania cambodica* [44].

Lignans and neolignans serve as chemotaxonomic markers for the genus *Licaria* and were identified as the major classes in *L. armeniaca* under various extraction conditions [3]. These compounds were extracted from the wood of the trunks and fruits using percolation methods with benzene, ethanol, and a mixture of ethanol and water (9:1) [4]. In contrast, these classes were not detected in the present study, which analyzed leaves, thin branches, and thick branches, revealing a predominance of alkaloids. Various factors, including tissue type, can influence the content of lignans and neolignans—since they are primarily located in woody tissues—environmental conditions, nutrition, and the maturity of the plant [45]. Additionally, the choice of solvents and extraction conditions significantly impacts the recovery of these metabolites. In this study, polar solvents such as ethanol and water were used, which may not be optimal for recovering lignans and neolignans [46,47].

**Figure 5:**
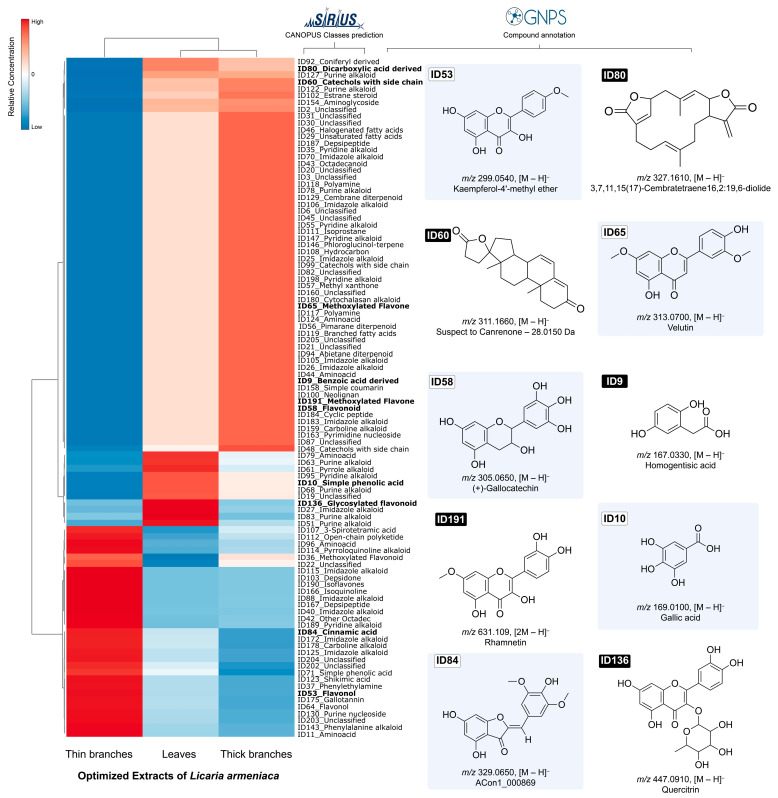
Heatmap containing the top 100 features with VIP scores > 1.5 extracted from pairwise PLS-DA models.

**Figure A1:**
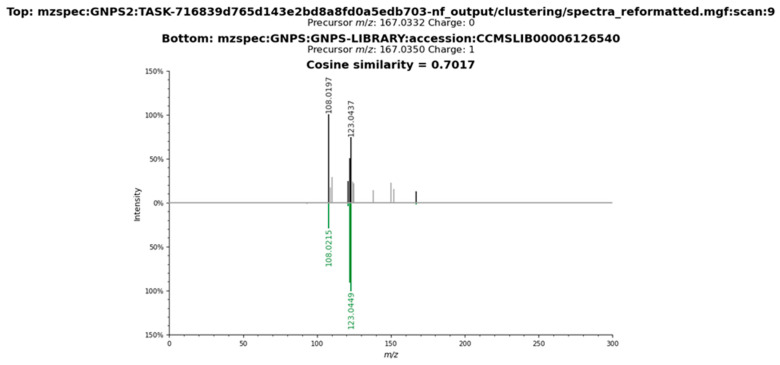
Mirror plot between experimental spectrum of *m*/*z* 167.0330, [M − H]^−^ and reference spectra of Homogentisic acid (annotated using the GNPS library, ID 9).

**Figure A2:**
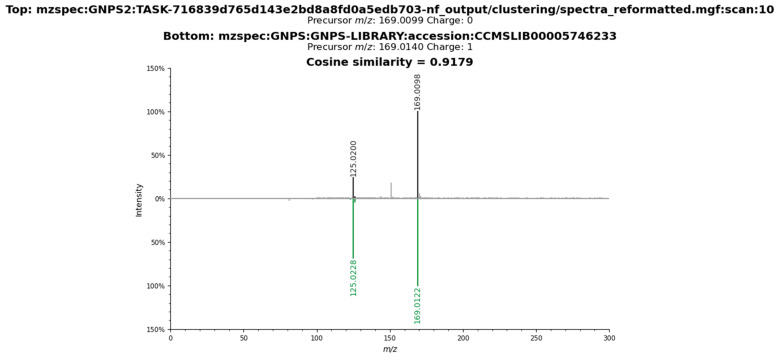
Mirror plot between experimental spectrum of *m*/*z* 169.0100, [M−H]^−^ and reference spectra of Gallic acid (annotated using the GNPS library, ID10).

**Figure A3:**
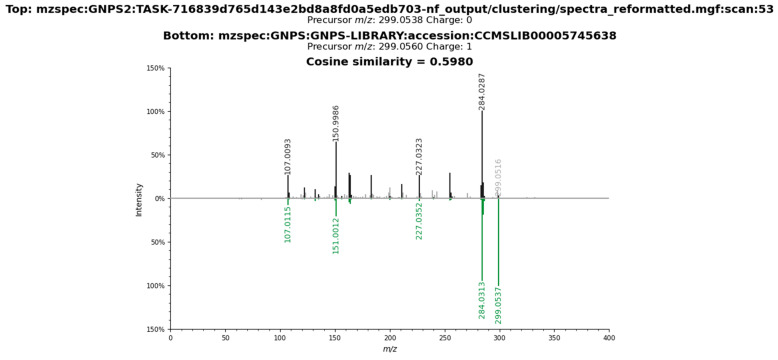
Mirror plot between experimental spectrum of *m*/*z* 299.0540, [M−H]^−^ and reference spectra of Kaempferol-4′-methyl ether (annotated using the GNPS library, ID53).

**Figure A4:**
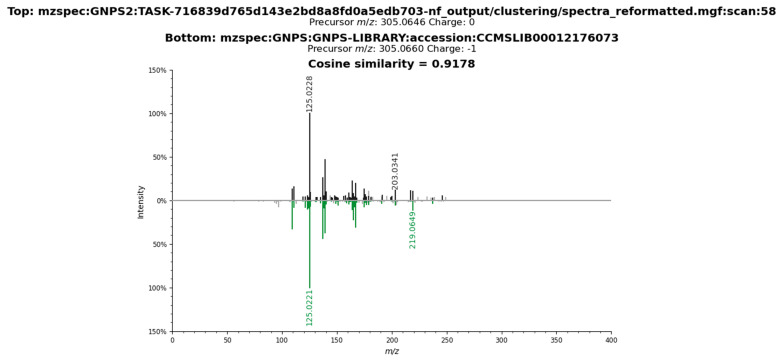
Mirror plot between experimental spectrum of *m*/*z* 305.0650, [M−H]^−^ and reference spectra of (+)-Gallocatechin (annotated using the GNPS library, ID58).

**Figure A5:**
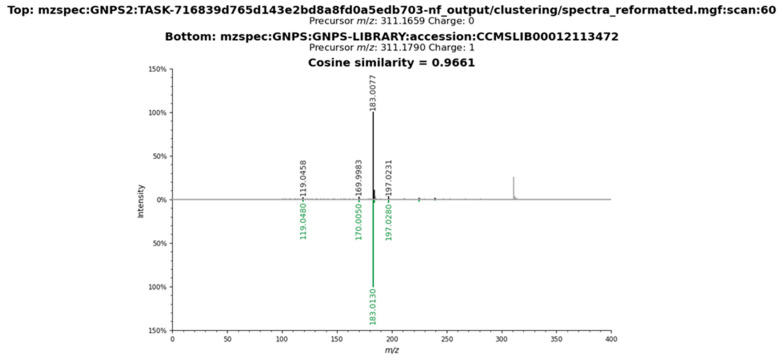
Mirror plot between experimental spectrum of *m*/*z* 311.1660, [M−H]^−^ and reference spectra suspect to Canrenone—28.0150 Da (annotated using the GNPS library, ID60).

**Figure A6:**
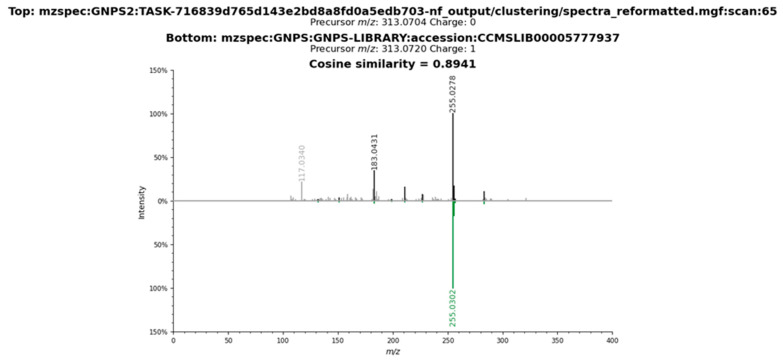
Mirror plot between experimental spectrum of *m*/*z* 313.0700, [M−H]^−^ and reference spectra of Velutin (annotated using the GNPS library, ID65).

**Figure A7:**
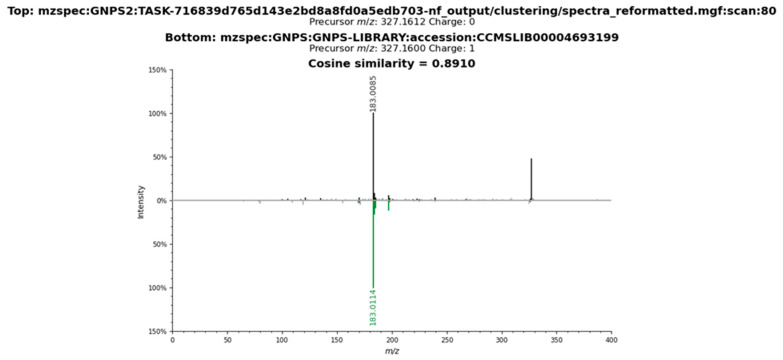
Mirror plot between experimental spectrum of *m*/*z* 327.1610, [M−H]^−^ and reference spectra of 3,7,11,15(17)-Cembratetraene16,2:19,6-diolide (annotated using the GNPS library, ID80).

**Figure A8:**
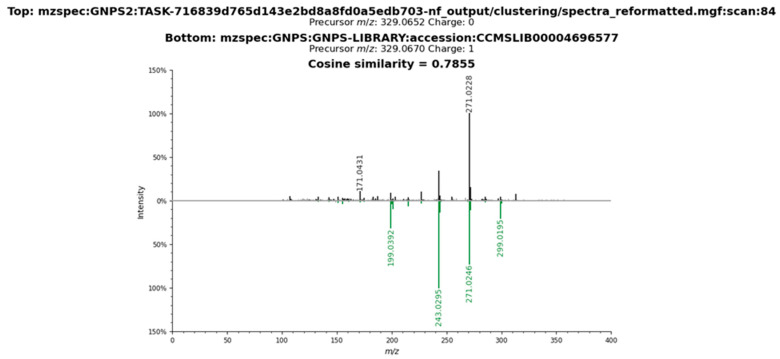
Mirror plot between experimental spectrum of *m*/*z* 329.0650, [M−H]^−^ and reference spectra of ACon1_000869 (annotated using the GNPS library, ID84).

**Figure A9:**
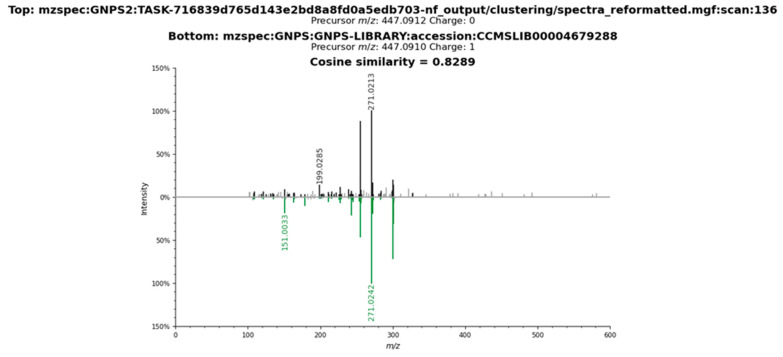
Mirror plot between experimental spectrum of *m*/*z* 447.0910, [M−H]^−^ and reference spectra of Quercitrin (annotated using the GNPS library, ID136).

**Figure A10:**
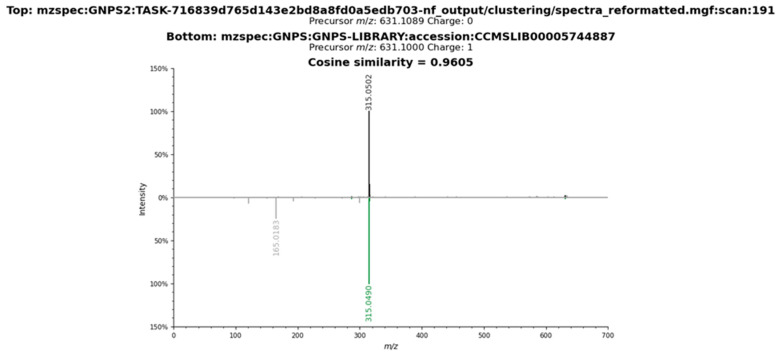
Mirror plot between experimental spectrum of *m*/*z* 631.109, [M−H]^−^ and reference spectra of Rhamnetin (annotated using the GNPS library, ID191).

Based on the Metabolomics Standards Initiative (MSI) putative annotations were obtained at level 2 (GNPS annotated compounds) and level 3 [48] through Sirius 4 [49], using the CANOPUS systematic classification [50]. This process enabled the annotation of 10 compounds (see Figure 5 and Appendix A.2 Figure A1, Figure A2, Figure A3, Figure A4, Figure A5, Figure A6, Figure A7, Figure A8, Figure A9 and Figure A10) from various classes of metabolites, with a particular emphasis on flavonoids and other phenolic compounds.

The identified flavonoids include velutin, rhamnetin, (+)-gallocatechin, quercitrin, and kaempferol-4′-methyl ether, all of which are known for their diverse pharmacological properties, such as antioxidant, anti-inflammatory, and antitumor effects [51]. Among these, quercetin—a glycosylated flavonol—demonstrates significant biological potential for treating metabolic bone diseases, gastrointestinal disorders, and cardiovascular and cerebrovascular conditions [52,53]. Among the phenolic compounds, homogentisic, gallic, and cinnamic acids stand out, and are widely investigated for their antioxidant, anticancer, anti-inflammatory, and antibacterial effects, as well as potential neuroprotective and hypoglycemic effects [54,55,56,57,58]. Furthermore, gallic and cinnamic acids and their derivatives present applications in the food and cosmetics industries [59,60,61,62].

The compound canrenone is a steroidal terpene and an active metabolite of spirolactone. Phytochemical studies have demonstrated anti-HIV activity in vitro in association with other terpenes and flavonoids from the ethanolic extract of *Artemisia campestri* (Asteraceae) [63].

The compound 3,7,11,15, (17)-cembratetraene 16,2:19,6-diolide is a cembrane-type diterpene that has been reported as ovatodiolide. It has primarily been isolated from *Anisomeles indica* (Lamiaceae) [64]. Pharmacological studies indicate that this compound exhibits in vitro antitumor activity, proving effective against glioblastoma, gastric cancer, and cervical cancer cells [65,66,67]. Additionally, it demonstrates anti-inflammatory and antibacterial properties [68,69].

These findings regarding the compounds in *L. armeniaca* enhance our understanding of the species as a potential source of bioactive natural compounds that could serve as candidates for new drugs.

### 2.4. Cytotoxic Activity

The cell availability was measured after 72 h, and the samples with an IC_50_ value < 100 μg/mL were considered relevant active [70]. All samples displayed good cytotoxicity against the cancer cell lines; however, with different values of the selective index (Table 3). Based on cytotoxicity against a normal cell line (Murine Macrophages RAW 264.7), the selective index (SI) for each sample was calculated. The SI value indicates differential activity, with higher SI values corresponding to increased selectivity. Conversely, an SI value below 2 is indicative of general compound toxicity [71]. The leaf extract was more selective to glioblastoma cell lines (IC_50_, 11.52 μg/mL, SI: 2.5) while thick branches showed high selective against gastric ascites (IC_50_, 13.59 μg/mL, SI: 3.74) and lung cancer (IC_50_, 16.95 μg/mL, SI: 3.0). Despite thin branches extracts showing high cytotoxic activity, their SI values were low (SI < 1.00).

Plant alkaloids are structurally diverse molecules that offer potential as sources for new drugs with cytotoxic and antiproliferative effects [72]. Recently, a study highlighted the importance of plant alkaloids in the treatment of glioblastoma (GBM), including two Lauraceae species (*Litsea glutinosa, Neolitsea konishii*). Most alkaloids act in a concentration-dependent manner by (1) reducing glioma cell viability, (2) suppressing proliferation, (3) inhibiting migration and invasion, (4) inducing cell death, (5) downregulating Bcl-2 and key signaling pathways, (6) exhibiting antiangiogenic effects, (7) reducing tumor weight, and (8) improving survival rates [73]. The imidazole skeleton affected the cell availability of four glioblastoma cell lines (U87, T98G, U87, G55T2) without discrimination, contributing to antineoplastic effects [74]. In contrast, three new pyridine-type alkaloids isolated from the aerial parts of *Vinca major* cultivated in Pakistan were evaluated for their cytotoxicity against glioblastoma cell lines (U-87MG and T98G) and lung cancer cell line A-549, but none of them was active at 20 μg/mL concentration [75]. Moreover, alkaloids show promise in treating gastrointestinal cancers due to their ability to influence cell signaling pathways. They can induce cell cycle arrest at G0/G1, S, and G2/M phases, as well as promote apoptosis. Additionally, they target key metabolic pathways, including p53, β-catenin, MAPK, and PIM3 [76].

## 3. Materials and Methods

### 3.1. Plant Material

*Licaria armeniaca* (Lauraceae) was collected in Utinga Camillo Vianna State Park Conservation Unit (1°24′45.1″ S, 48°24′11.6″ W) Belém, Pará, Brazil in December/2023 and deposited at the Emílio Goeldi Museum Herbarium, in Belém, Pará registered by voucher MG253515. The research project was registered on the National System for Governance of Genetic Heritage and Associated Traditional Knowledge (SisGen A94E1B6). The botanical material was dried in a ventilation oven for 48 h and ground in a knife mill. Approximately 1.182 kg of plant material was obtained, distributed in thin branches (318 g), thick branches (594 g) and leaves (269 g).

### 3.2. Ultrasound-Assisted Extraction

The extracts were obtained by the ultrasound-assisted extraction (UAE) method (Ultrasonic bath—7Lab, SSBu model; 3.8 L; 100 W). Seventeen tests were performed. The conditions for the ethanol percentage in water, extraction time and solid–liquid ratio followed the values defined in the CCRD matrix (Appendix A.1). The extracts obtained were filtered and the remaining solvents were evaporated using the Rotary Evaporator (QUIMIS, Q344M), ending with freeze-drying.

### 3.3. Experimental Design and Optimization

The Central Composite Rotational Design (CCRD) was applied to determine the best combination of controllable variables in the extraction of bioactive compounds from *L. armeniaca*. The input variables were selected based on studies described in the literature: [27,77] ethanol percentage (X_1_), extraction time (X_2_), solid/liquid ratio (X_3_), with their actual and coded levels described in Table 4. The liquid–solid ratio for the sheets was adjusted to obtain better working conditions.

According to the matrix of a DCCR with 3 variables, a total of 17 tests were performed, distributed in 8 factorial points (2^3^), 6 axial points (2 × 3) and 3 replicates of the central point (Appendix A.1) [78]. The tests were performed in random order.

For the response variables, the antioxidant activity was defined (Y_1_^F^) in percentage of inhibition of the free radical DPPH and the concentration of total phenolic compounds (Y_2_^GF^), in milligram of gallic acid equivalent per gram of extract (mgEAG/g).

### 3.4. Antioxidant Activity

The antioxidant activity of the extracts was evaluated by the DPPH radical scavenging method [79]. The extracts were solubilized in methanol (20 mg/mL). Aliquots of the solution (50 µL) were mixed with 1950 µL of DPPH solution; the negative control consisted of replacing the sample with the solvent methanol, and all were carried out in triplicate. The test was carried out on an LMR 96 Microplate Reader (Loccus, São Paulo, Brazil), with a standard 96-position microplate, flat bottom. The absorbance reading was taken at a wavelength of 520 nm. Antioxidant activity was expressed by the percentage of inhibition of free radicals (I %).

### 3.5. Total Phenolics

The total phenolics concentration (TPC) in extracts was determined according to the Folin–Ciocalteu procedure [79,80]. The extracts were solubilized in methanol at an initial concentration of 20 mg/mL and diluted with water. Aliquots (500 µL) of the aqueous solution were mixed with 250 µL of Folin–Ciocalteu reagent (1.0 N) and 1250 µL of sodium carbonate (75 g/L). The absorbance was measured after 30 min at 760 nm and 25 °C (UV–Vis Ultrospec 5300 Pro—Amersham Biosciences, Buckinghamshire, UK). The experimental calibration curve was prepared in triplicate using gallic acid at concentrations of 2.0, 4.0, 8.0, 16.0, 24.0, 32.0 and 40.0 mg/L corresponding to absorbance values of 0.04 ± 0.00, 0.11 ± 0.01, 0.24 ± 0.01, 0.44 ± 0.01, 0.64 ± 0.01, 0.87 ± 0.0, 1.05 ± 0.01, respectively (R^2^ = 0.998), submitted for the same procedure. The total phenolics content was expressed as gallic acid equivalents (GAE) in milligrams per gram of extract (mg GAE/g).

### 3.6. Statistical Analysis and Experimental Validation

The results obtained were analyzed with the help of Statistics 7.0 and Excel 16 softwares. For the predicted responses Y_1_ and Y_2_, the second-order polynomial equation model Equation (3) was considered:

Y = β_0_ + β_1_X_1_ + β_2_X_2_ + β_3_X_3_ + β_11_X_1_^2^ + β_2_^2^X_2_^2^ + β_3_^3^X_3_^2^ + β_1_^2^X_1_X_2_ + β_1_^3^X_1_X_3_ + β_2_^3^X_2_X_3_ (3)

where β_0_ is the intercept, β_1_, β_2_ and β_3_ are the linear coefficients, β_11_, β_22_ and β_33_ are the quadratic coefficients and β_12_, β_13_ and β_23_ are the interaction coefficients.

Using the regression coefficient table, the parameters with a significant effect on each response were selected (*p* ≤ 0.1). The variables were used in the construction of a mathematical equation. Analysis of Variance (ANOVA) associated with the regression coefficient (R^2^), Fischer’s test and lack of fit (LOF) were used to determine whether the model was significant and predictive.

Using Statistica 7.0 software, response surfaces (RSM) were generated to graphically demonstrate the behavior between the independent variables and the responses. The determination of the optimum points was done by solving the final equation and was adapted from the RSM analysis and the desirability approach. To validate the proposed mathematical model, the relative error (E%) between the predicted and experimental responses was evaluated. Thus, the optimum points were reproduced in triplicate and subjected to analyses of total phenolic content and antioxidant activity, following the procedures already described. The relative error was also calculated.

### 3.7. Calculation of Global Desirability of Harrington (d)

The calculation of the global desirability function followed methodology of Harrington [24]. Desirability (d) followed the following criterion:

Value (d) 0.8–1.00—Quite acceptable;
Value (d) 0.63–0.79—Acceptable;
Value (d) 0.37–0.62—Satisfactory;
Value (d) 0.0–0.36—Unacceptable.

### 3.8. Liquid Chromatography–Tandem Mass Spectrometry Analysis

The liquid chromatography–tandem mass spectrometry (LC-MS/MS) analysis was carried out using a Xevo G2-S QTof high-resolution mass spectrometer (Waters Corp., Milford, MA, USA) equipped with a Lockspray source. Leucine-enkephalin served as the reference compound to ensure accurate mass measurements. System control and data acquisition were performed using MassLynx 4.1 software. Triplicates of 2 μL aliquot from each extract, prepared at a concentration of 1000 ppm (1 mg/mL), were injected and separated using a BEH C18 column (Waters Corp.; 50 mm × 2.1 mm; 1.7 μm particle size) maintained at 40 °C. The mobile phase consisted of ultrapure water (solvent A) and acetonitrile (solvent B), with a flow rate of 300 μL/min. The chromatographic run lasted 15 min, following this gradient: 0–10 min (linear increase from 5% to 95% B), 10.01–12.0 min (column washing), 12.01–14.0 min (linear decrease from 95% to 5% B), and 14.01–15.0 min (maintained at 5% B). Data acquisition was performed in negative ionization (NI) mode within a mass range of *m*/*z* 50–1500. A Data-Dependent Acquisition (DDA) approach was used, applying a centroid format, and selecting the five most intense ions (Top 5 Experiment). The collision energy was varied between 15 and 45 eV. The scan time was set to 0.1 s, with a charge state of +2, a tolerance window of ±0.2 Da, and a peak extraction tolerance of 2 Da. Deisotoping parameters included a tolerance of ±3 Da and an extraction tolerance of 6 Da. The source and desolvation temperatures were maintained at 150 °C and 300 °C, respectively. The cone gas and desolvation gas flow rates were adjusted to 50 L/h and 800 L/h, respectively. The capillary voltage was set at 3.0 kV, and the cone voltage was 40 V. The LC-MS/MS raw data are publicly available at https://massive.ucsd.edu/ProteoSAFe/dataset.jsp?task=d0f22bcaf70546c8ad90ffcb6b03fec2 (accessed on 27 July 2025) [81].

### 3.9. Spectrometric Data Processing

Mass spectrometry data files obtained from the Xevo G2-S QTof instrument for all extracts were converted to the mzXML format using MSConvert 3.0.2 (ProteoWizard, Palo Alto, CA, USA) [82]. Data processing was carried out with MZmine version 4.0 [83]. Precursor and fragment intensity thresholds were set at 1.0 × 10^3^ (MS1) and 8.0 × 10^1^ (MS2), respectively. The total ion chromatograms (TICs) were generated using the ADAP algorithm. Retention times were considered within a range of 0.02 to 10.00 min, with a minimum group size of 3 and an intensity threshold of 1.0 × 10^3^. The maximum and minimum intensity values were established at 3.0 × 10^4^, and the scanner accuracy was configured at 0.002 *m*/*z* or 10 ppm. Chromatogram deconvolution was performed using the Local Minimum Resolver algorithm, applying an 80% chromatographic threshold. The minimum search interval for retention time/mobility was 0.050, with a precursor tolerance of 0.002 *m*/*z* or 10 ppm. Noise threshold settings were 15, while the absolute minimum height was 1.0 × 10^4^. The peak-to-peak ratio was set to 1.7, the peak duration ranged from 0.10 to 1.00, and the wavelength range (RT) was 0.05 to 0.15. Isotope detection was based on a peak window tolerance of 0.002 *m*/*z* or 10 ppm, a retention time tolerance of 0.2 min, and a maximum charge of 2. Peak alignment was conducted with an *m*/*z* tolerance of 3:1 per retention time and a retention time tolerance of 0.2 min. After alignment, the resulting data were filtered to eliminate duplicates and entries without an associated MS2 spectrum. The processed files were then exported in mgf and CSV formats for further analysis on the GNPS platform [84]. All spectra within the molecular networks were matched against reference spectra from the GNPS spectral libraries [84], using a cosine score threshold of 0.5 and requiring at least three (3) MS^2^ fragment matches. In addition, the Suspect Library [85] was consulted, and the corresponding job is accessible at the following link https://gnps2.org/status?task=3972b3df78414727bf8f5ed403600d68# (accessed on 20 May 2025). Additionally, CANOPUS [49], integrated into SIRIUS [50] was employed for the prediction of chemical classes. Statistical analyses were created via Jupyter Notebook (all codes are available at https://github.com/pwpgomes1, accessed on 27 July 2025).

### 3.10. Cytotoxic Activity by MTT

The cell viability was determined by reduction of the yellow dye 3- (4,5-dimethyl-2-thiazol)-2,5-diphenyl-2H-tetrazolium bromide (MTT) to a blue formazan product as previously described by Mosmann 1983 [86,87]. Plant tissue extracts were dissolved in DMSO at concentrations of 100, 50, 25, 12.5, 6.25, 3.13 and 1.56 μg/mL and then were tested for cytotoxic activity against the cancer cell lines AGP-01 (malignant gastric ascites), AHOL (Human glioblastoma) and A549 (lung cancer) as well as, against the normal human murine macrophages (RAW 264.7) cells. All cell lines were maintained in DMEM (Dulbecco’s Modified Eagle Medium) supplemented with 10% fetal bovine serum, 2 mM glutamine, 100 U/mL penicillin, 100 μg/mL streptomycin, at 37 °C and with 5% CO_2_. The DMSO final concentration in the culture medium was kept constant (<0.1%). Cells were plated in a 96-well plate (100 μL/well) at a concentration of 5 × 10^3^ cells/well. The samples were incubated again with the cells for a period of 72 h. The negative control received the same amount of DMSO (0.001% at the highest concentration. Cytotoxic activity was identified using a multiwell scanning spectrophotometer set to a wavelength of 570 nm.

## 4. Conclusions

The utilization of statistical experimental design has provided an in-depth analysis of ultrasound-assisted extraction of *L. armeniaca* plant tissues. The findings reveal that this method is not only highly effective for extracting valuable alkaloids and phenolic compounds. Through this process, we successfully optimized extraction conditions, pinpointing the ideal parameters using the desirability function: for leaf extracts, the % DPPH inhibition (Ethanol 64.9%, t: 26.1 min, 6.23% *m*/*v*) and TPC for the thin branches (Ethanol 73.8%, t: 31.3 min, 11.0% *m*/*v*). For thick branches, its ideal conditions were based on the best results of DPPH and TCP (Ethanol 50.0%, t: 35.0 min, 11.0% *m*/*v*). Furthermore, the integration of machine learning and mirror graphs in the GNPS library for compound annotation has proven to be an exceptionally effective strategy, uncovering crucial insights into the chemical classes, subclasses, and bioactive natural compounds present in the leaves, thin twigs, and thick twigs of *L. armeniaca*. Alkaloids were predominant in leaf extracts (46.34%), thin branches (35.97%) and thick branches (32.14%), followed by amino acids and peptides (19.51%, 20.86% and 25.0%, respectively). All extracts displayed significant cytotoxic activity (IC_50_ < 100 μg/mL) with different selective index values. Regarding the cytotoxicity against normal cells (RAW 264.7), the thick branches extract displayed low toxicity. This innovative approach highlights the potential of this extraction technique to advance our understanding of plant-based bioactive compounds.

## Figures and Tables

**Table 1:** Analysis of variance (ANOVA) and F test for planning experiments on ultrasound-assisted removal of phenolic compounds de *L. armeniaca*.

Source of Variation	QS	Df	MQ	F_calc._	F_tab. (*p* ≤ 0.1)_
Leaves—DPPH					
Regression	2515.1	8	314.4	14.20	2.59
Residue	177.1	8	22.1		
Lack of fit	170.710	6	28.452	8.92	9.33
Pure error	6.383	2	3.191		
Total	2692.234	16			
Adjusted R^2^	0.9342				
R^2^	0.9976				
Thin Branches—TPC					
Regression	26343.4	3	8781.1	12.23	2.56
Residue	9335.7	13	718.1		
Lack of fit	9034.92	11	821.36	5.46	9.4
Pure error	300.81	2	105.40		
Total	35,679.12	16			
Adjusted R^2^	0.7383				
R^2^	0.9916				
Thin Branches—DPPH					
Regression	582.0	8	72.8	1.47	2.59
Residue	397.1	8	49.6		
Lack of fit	394.0452	6	65.6742	42.61	9.33
Pure error	3.0824	2	1.5412		
Total	979.1334	16			
Adjusted R^2^	0.5944				
R^2^	0.9969				

**Table 2:** Predicted values obtained with the aid of the Desirability tool and observed values of the response variables DPPH radicals (Y_1_^F^) and total phenolic compounds (Y_2_^GF^). ^a^ Confidence interval at 95%.

Assays	Y_1_^F^ (%)	Y_2_^GF^ (mgGAE/g)
Predicted	63.48	291.30
Observed	61.16–64.02 ^a^	275.4–315.9 ^a^
MAE (%)	1.78	3.33

MAE: Mean Absolute Error.

**Table 3:** Cytotoxic activity of the *Licaria armeniaca* extracts on cell lines.

Extract	Cell lines IC_50_ (µg/mL)			
	AGP-01 Gastric Ascite	AHOL Human Glioblastoma	A549 Lung Cancer	RAW 264.7 Murine Macrophages
Leaves	27.63 (20.82–36.67) SI: 1.04	11.52 (8.054–16.49) SI: 2.5	45.97 (14.87–142.1) SI: 0.63	28.81 (23.17–35.83)
Thin branches	15.71 (12.22–20.22) SI: 0.94	18.89 (13.55–26.36) SI: 0.78	31.5 (19.67–50.46) SI: 0.47	14.72 (10.93–19.84)
Thick branches	13.59 (10.29–17.96) SI: 3.74	26.52 (23.24–30.26) SI: 1.92	16.95 (12.92–22.24) SI: 3.0	50.86 (43.24–59.83)

**Table 4:** The DCCR experimental design with the independent variables (actual and coded values).

Variable		Variable Level				
		−1.68	−1	0	1	1.68
X_1_	EtOH percentage (%)	0	20.24	50	79.76	100
X_2_	Extraction time (min)	5	17.14	35	52.86	65
X_3_	Solid-to-liquid ratio to leaves (% *m*/*v*)	0.8	2.24	4.4	6.54	8.0
X_3_	Solid-to-liquid ratio for branches (% *m*/*v*)	2	5.56	11	16.35	20

## Data Availability

The original contributions presented in this study are included in the article. Further inquiries can be directed to the corresponding author.

## Abbreviations

UAE	Ultrasound-Assisted Extraction
CCRD	Central Composite Rotational Design
RSM	Response Surface Methodology
LC-MS/MS	Liquid Chromatography coupled with Tandem Mass Spectrometry
GNPS	Global Natural Products Social Molecular Networking
TPC	Total phenolics
DPPH	2,2-diphenyl-1-picrylhydrazyl
SLR	Solid-to-liquid ratio
EtOH	Ethanol
MAE	microwave-assisted extraction
MSI	Metabolomics Standards Initiative
IC_50_	Inhibitory Concentration 50%
SI	Selective index
SiSGen	National System for Governance of Genetic Heritage and Associated Traditional Knowledge
ANOVA	Analysis of Variance
R^2^	Regression coefficient
LOF	Fischer’s test and lack of fit

## Appendix A

### Appendix A.1. CCRD Planning Matrix

	**Level of Independent Variables**			
**Run**	**% EtOH**	**t (min)**	**RSL (% *m*/*v*)**	
			**Leaves**	**Branches**
1	20.24 (−1)	17.14 (−1)	2.24 (−1)	5.56 (−1)
2	79.76 (1)	17.14 (−1)	2.24 (−1)	5.56 (−1)
3	20.24 (−1)	52.86 (1)	2.24 (−1)	5.56 (−1)
4	20.24 (−1)	17.14 (−1)	1.12 (1)	16.35 (1)
5	79.76 (1)	52.86 (1)	2.24 (−1)	5.56 (−1)
6	79.76 (1)	17.14 (−1)	6.54 (1)	16.35 (1)
7	20.24 (−1)	52.86 (1)	6.54 (1)	16.35 (1)
8	79.76 (1)	52.86 (1)	6.54 (1)	16.35 (1)
9	0 (−1.68)	35 (0)	4.4 (0)	11 (0)
10	100 (1.68)	35 (0)	4.4 (0)	11 (0)
11	50 (0)	5 (−1.68)	4.4 (0)	11 (0)
12	50 (0)	65 (1.68)	4.4 (0)	11 (0)
13	50 (0)	35 (0)	0.8 (−1.68)	2 (−1.68)
14	50 (0)	35 (0)	8 (1.68)	20 (1.68)
15	50 (0)	35 (0)	4.4 (0)	11 (0)
16	50 (0)	35 (0)	4.4 (0)	11 (0)
17	50 (0)	35 (0)	4.4 (0)	11 (0)
